# Arabidopsis TIC236 contributes to proplastid development and chloroplast biogenesis during embryogenesis

**DOI:** 10.3389/fpls.2024.1424994

**Published:** 2024-08-23

**Authors:** Mei Liu, Lifen Chen, Shijie Gu, Aiwei Zhang, Mengjuan Tong, Shuailei Wang, Juntao Wang, Yirui Zhu, Jingsheng Zhang, Yu Sun, Yi Guo, Rui Li

**Affiliations:** ^1^ Hebei Research Center of the Basic Discipline of Cell Biology, Hebei Normal University, Shijiazhuang, China; ^2^ Ministry of Education Key Laboratory of Molecular and Cellular Biology, Hebei Normal University, Shijiazhuang, China; ^3^ Hebei Collaboration Innovation Center for Cell Signaling and Environmental Adaptation, Hebei Normal University, Shijiazhuang, China; ^4^ Hebei Key Laboratory of Molecular and Cellular Biology, Hebei Normal University, Shijiazhuang, China; ^5^ College of Life Sciences, Hebei Normal University, Shijiazhuang, China

**Keywords:** proplastid, chloroplast, TIC236, embryo development, Arabidopsis

## Abstract

Plastids are essential, semi-autonomous organelles in plants that carry out a multitude of functions during development. Plastids existing in different subtypes are derived from proplastids progenitors and interconvert in response to environmental and growth cues. Most efforts focus on the differentiation from proplastid to other forms. However, the studies of proplastid development are insufficient and whether proplastid biogenesis affects plant growth is yet to be determined. Arabidopsis TIC236, a translocon component at the inner membrane of the chloroplast envelope, is critical for importing chloroplast-targeted preproteins and chloroplast division. In this study, we uncovered the fundamental influence of proplastid biogenesis on embryo development by exploring the function of TIC236 during embryogenesis. Widespread and strong expression of *TIC236* was observed in leaves and embryos. The null mutant *tic236* had an embryo-lethal phenotype, with cell division in the mutant embryos delayed starting at the octant stage and arrested at the globular stage. Transmission electron microscopy revealed enlarged proplastids with an aberrant inner structure at the dermatogen and globular stages that ultimately did not differentiate into chloroplasts. Additionally, the fluorescence signal distribution patterns of *tic236* embryos carrying the *pDR5rev::3xVENUS-N7*, *pPIN1::PIN1-GFP*, *pWOX5::GFP*, and *pSCR::H2B-YFP* reporter systems were altered. Together, we provide genetic evidence supporting proplastid biogenesis plays a vital role in embryo development and TIC236 is identified as an indispensable player, ensuring normal proplastid development.

## Introduction

Plastids are important semi-autonomous organelles in plants that are involved in photosynthesis and the biosynthesis of amino acids, lipids, nucleotides, and hormones (Sakamoto et al., 2008). Plastids exist as different subtypes, including chloroplasts, starch-rich amyloplasts, chromoplasts, elaioplasts, and leucoplasts, which participate in diverse physiological processes during plant growth and development (Liebers et al., 2017). Plastids are derived from proplastid progenitors. Undifferentiated proplastids can differentiate into four major forms: etioplasts, chloroplasts, chromoplasts, and amyloplasts. A variety of forms interconvert into each other relying on environmental and developmental cues (Choi et al., 2021). However, most research on proplastids has focused on its differentiation into other forms of plastids. The knowledge of biogenesis and metabolism of proplastids is very limited (Moller, 2004).

Proplastids are mainly distributed in reproductive cells, embryos, seeds, and meristematic tissues (Sierra et al., 2023); therefore, it is difficult to observe and isolate these small and colorless organelles. Most research on proplastids is based on electron microscopic observations of meristematic tissue (Moller, 2004). Proplastids in meristematic tissue are devoid of pigments, have few internal vesicles, and lack obvious thylakoid structures (Sakamoto et al., 2008). Meanwhile, the metabolic function of proplastids is under debate. One opinion is that proplastids have no metabolic function, except for differentiating into other types of plastids (Gajecka et al., 2020). Others state that proplastids, though undifferentiated, supply fatty acid, amino acid, and nucleotide precursors for plant cells. A proteomic and transcriptional analysis of the envelope membranes of proplastids indicated that proplastids are active organelles (Bräutigam and Weber, 2009; Dalal et al., 2018). Proplastids may import precursor metabolites and export products for cells (Bräutigam and Weber, 2009). Accumulation and replication of chloroplasts (ARC6) is required for proplastid division in meristematic tissues. Accumulation and replication of chloroplasts 6 (ARC6) is required for proplastid division in meristematic tissues. But whether enlarged proplastids in *arc6* mutants lead to defects in shoot apical meristems, however, remains to be determined (Pyke et al., 1994; Robertson et al., 1995). Thus, genetic evidence is still lacking to support the function of proplastid development.

Embryonic proplastids are the progenitor of plastids in higher plant cells (Jarvis and López-Juez, 2013). Embryogenesis in plants proceeds through several important stages (zygote, 1-cell, 2/4-cell, octant, dermatogen, globular, heart, torpedo, and cotyledon) to form a mature embryo (ten Hove et al., 2015). Proplastids begin to differentiate into chloroplasts at the late globular stage (Mansfield, 1991), which is critical for normal embryo development (Hsu et al., 2010; Yadav et al., 2019). Substantial amount of embryo-lethal mutants display impaired differentiation from proplastid into chloroplast (Bryant et al., 2011; Trösch and Jarvis, 2011; Meinke, 2020). About 30% of Arabidopsis genes which are essential for embryogenesis, are associated with plastid development (Hsu et al., 2010; Meinke, 2020). Recently, chloroplast-located Arabidopsis EMB2726, and maize EMB27 are found to be required for proplastid differentiation and embryo development (Li et al., 2021; Liu et al., 2024). A plastid-targeted ankyrin repeat protein (AKRP) regulates gametophyte and early embryo development in Arabidopsis (Kulichová et al., 2022). However, whether proplastids play important roles before they differentiate into chloroplasts during embryonic development is poorly understood.

Plastids evolved from Gram-negative cyanobacterial endosymbionts whose genes were mostly transferred to the host’s nuclear genome (Fang et al., 2022). Plastids have a two-membrane envelope comprised of outer and inner membranes. Most plastid proteins are encoded in the nuclear genome, translated in the cytoplasm, and then transported into plastids through translocons at the outer- and inner-envelope membranes (TOC and TIC, respectively) (Fang et al., 2022). TIC236 is a TIC component in Arabidopsis located in the inner envelope of chloroplasts (Chuang et al., 2021). TIC236 has a 230-kDa domain that projects into the intermembrane space and interacts directly with the core TOC component TOC75 connecting the inner- and outer-membrane translocons (Chen et al., 2018). This creates a stable backbone to facilitate the translocation of chloroplast preproteins into the stroma. TOC75 and TIC236, homologs of BamA and TamB5 (bacterial outer- and inner-membrane translocons, respectively), occur only in plants and co-evolved during plant evolution (Chen et al., 2018). But recently TIC236 is not identified in cryoelectron microscopy structure of TOC-TIC supercomplex in *Chlamydomonas* (Jin et al., 2022; Liu et al., 2023). Thus, the role of TIC236 in translocating preproteins into chloroplast was challenged. Moreover, TIC236 may also control chloroplast size by importing proteins involved in chloroplast division (Fang et al., 2022). Homozygous mutants of a weak *tic236* allele have enlarged chloroplasts in their leaves, whereas null mutants of TIC236, originally referred to as *embryo-defective 2410*, show embryonic lethality via an unknown molecular mechanism (Tzafrir et al., 2004; Fang et al., 2022). Additionally, TIC236 homologs, including substandard starch grain4 (SSG4) in rice and defective kernel5 (DEK5) in maize, have been shown to regulate chloroplast size in leaves and starch grain size in the amyloplasts of the endosperm (Matsushima et al., 2014; Zhang et al., 2019).

In this study, we explored the function of TIC236 in embryo development. *tic236* null mutants showed delayed cell division at the octant stage and asynchronous division at subsequent stages, with an arrest in embryo development at the late globular stage. Transmission electron microscopy (TEM) revealed that TIC236 maintains the normal morphology and function of proplastids and that it promotes the differentiation of proplastids to chloroplasts during early embryo development. Together, our data provide genetic evidence for the important role of proplastid biogenesis in early embryonic development.

## Results

### *TIC236* is strongly expressed in embryos, seedlings, and leaves

Arabidopsis TIC236, which includes a plastid-targeting transit peptide, an N-terminal transmembrane domain, and a C-terminal DUF490 domain, is a conserved inner-membrane translocon that helps import proteins into chloroplasts (Chen et al., 2018; Fang et al., 2022). Initially, TIC236 was reported to be essential for embryo development by the Seed Genes Project (Tzafrir et al., 2003). However, how *TIC236* regulates embryo development is unclear.

**Figure 1:**
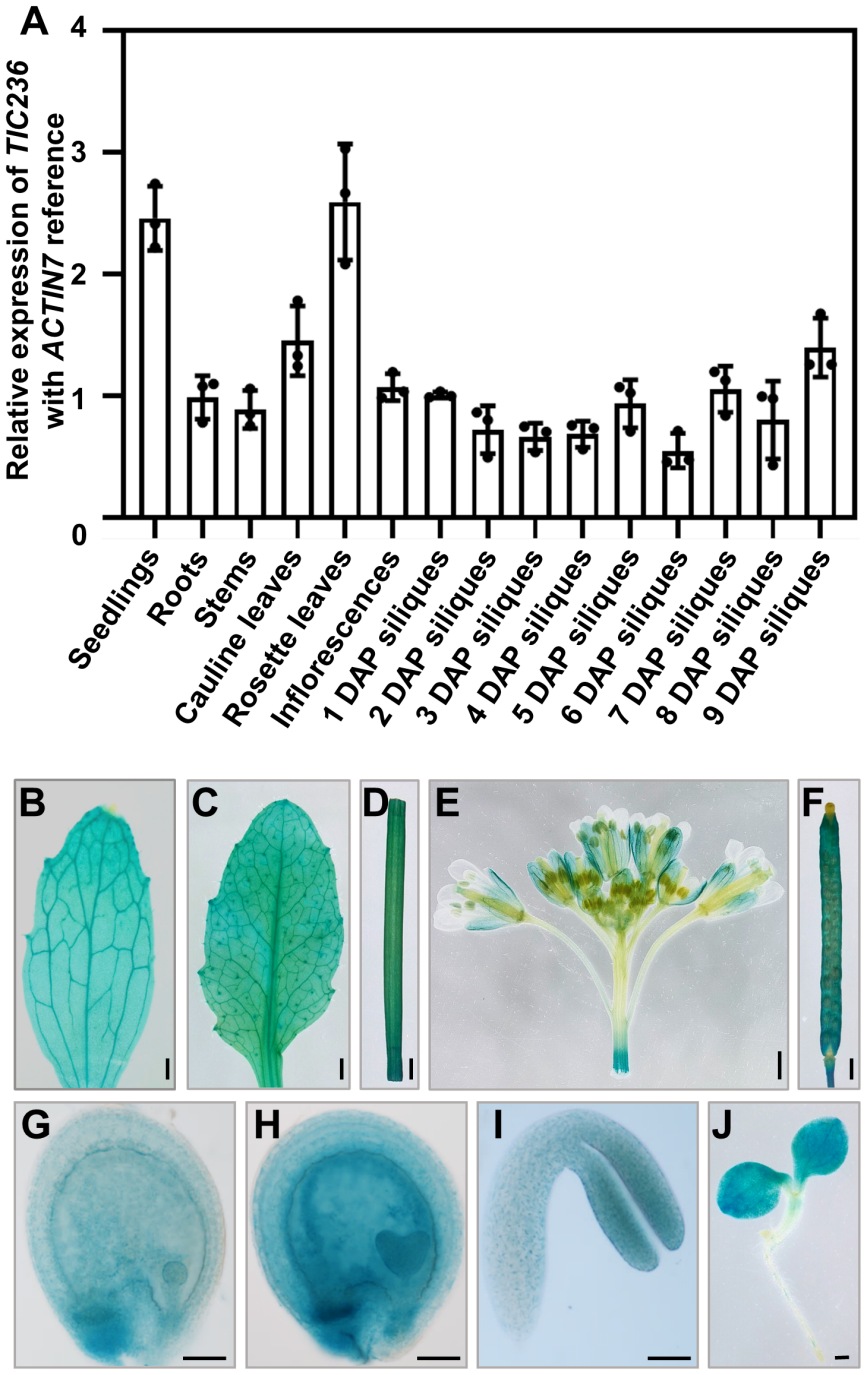
Expression profile analysis of *TIC236*. **(A)** Relative expression of *TIC236* in different tissues as revealed by qRT-PCR*. ACTIN7* was used as an internal control. Error bars indicate the standard error. DAP, day after pollination. **(B–J)** GUS staining of cauline leaves **(B)**, rosette leaves **(C)**, stem **(D)**, inflorescences **(E)**, siliques **(F)**, globular embryo **(G)**, heart embryo **(H)**, cotyledon **(I)** and seedlings **(J)** from *Pro_TIC236_:GUS* transgenic plants. Bars = 1 mm for **(B–F, J)**; 50 μm for **(G–I)**.

To investigate the detailed functions of *TIC236* in plant growth, the expression profiles of *TIC236* were explored. The expression map visualized in an Electronic Fluorescent Pictograph (eFP) browser (http://bar.utoronto.ca/eplant/; Nakabayashi et al., 2005; Schmid et al., 2005; Winter et al., 2007) revealed that *TIC236* is widely expressed with a higher level in seedlings, leaves, and embryos (**Supplementary Figure 1**). Then, the expression pattern of *TIC236* was tested by quantitative reverse transcription-PCR (qRT-PCR). RNA from various tissues, including seedlings, roots, stems, cauline leaves, rosette leaves, inflorescences, and siliques at 1–9 days after pollination (DAP), were extracted and reverse-transcribed to cDNA. *TIC236* was expressed ubiquitously in all of the tissues analyzed, with higher expression levels in rosette leaves, cauline leaves, and seedlings. The cells of these tissues were rich in chloroplasts, consistent with the function of TIC236 in chloroplasts. In the siliques, the highest level of expression was detected in materials collected at 9 DAP (**Figure 1A**).

To analyze *TIC236* expression further, the putative promoter (2054 bp upstream of the start codon [ATG]) of *TIC236* was fused to the β-glucuronidase (GUS) reporter gene. *Pro_TIC236_::GUS* transgenic plants were generated in a wild-type (WT) background and the expression profile of *TIC236* was assessed by GUS staining. In vegetative tissues, GUS signals were widely detected in seedlings, cauline leaves, rosette leaves, and stems (**Figures 1B–D, J**). In reproductive tissues, GUS was expressed in inflorescences—mainly in sepals, filaments, stigmas, and siliques (**Figures 1E, F**). During embryo development, GUS staining was observed in globular, heart, and cotyledon stage embryos, with the strongest signals at the heart stage (**Figures 1G–I**). Together, these results show that *TIC236* is strongly and ubiquitously expressed in leaves, seedlings, and developing embryos.

### Genomic *TIC236* rescues the embryo lethality of *tic236*

**Figure 2:**
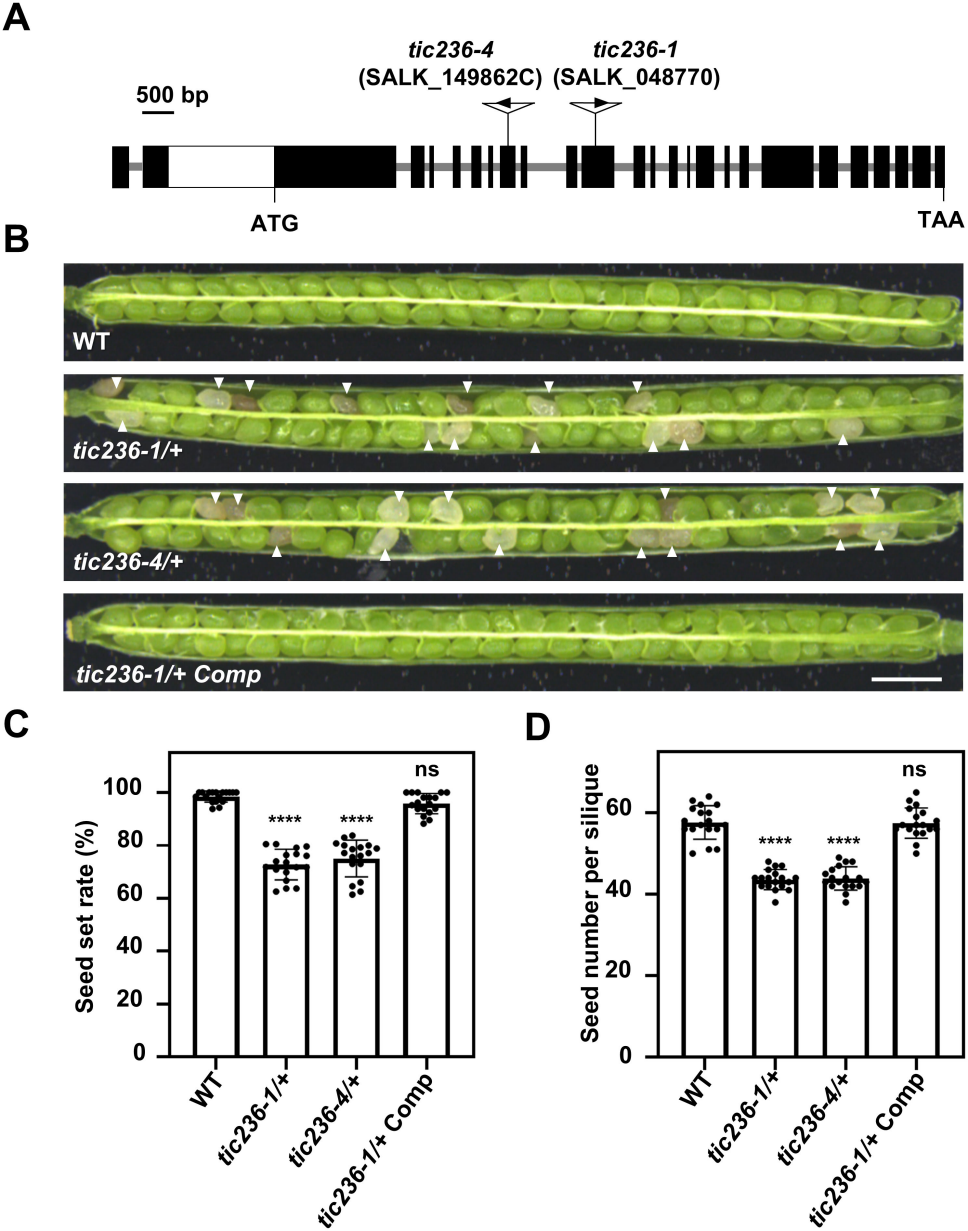
Characterization of the *tic236* mutants and complementation lines. **(A)** Schematic diagram of the *TIC236* genomic sequence structure and each T-DNA insertion site. Black boxes and grey lines indicate exons and introns, respectively. The white box indicates the untranslated region. **(B)** Seed development in wild type, *tic236-1/+*, *tic236-4/+* and functionally complemented *tic236-1/+* transgenic plants. The arrow heads show the aborted white seeds. Bar = 1 mm. **(C, D)** Statistical analysis of the seed setting and seed number from genotypes as shown in **(B)**. Results shown are means ± SD (n = 18). Asterisks indicate significant difference relative to wild type (Student’s t test, ****P < 0.0001).

To delve further into the biological function of *TIC236* in plant development, two T-DNA insertion mutants, *tic236-1* (SALK_048770) and *tic236-4* (SALK_149862C), were obtained from the Arabidopsis Biological Resource Center (Alonso et al., 2003). The mutant alleles *tic236-1*, *tic236-2*, and *tic236-3* were described previously (Chen et al., 2018; Fang et al., 2022), whereas *tic236-4* is a new allele. The full-length genomic sequence of *TIC236* contains 10,935 bases comprising 24 exons and 23 introns. Two T-DNA insertions were located in exons 10 and 7, respectively (**Figure 2A**). Genotypic analysis of the *tic236/+* progeny revealed that only heterozygotes were obtained—no homozygotes were detected (**Supplementary Figures 2A, B**). In addition, white ovules were found in the siliques of the heterozygous mutants at a frequency of approximately 25% and the normal seed number in the *tic236/+* mutants was 25% lower than that in wild type (**Figures 2C, D**). Consistent with previous findings, this suggests that a homozygous *tic236* mutation is lethal (**Figures 2B–D**).

Next, segregation ratio analyses of the self-pollinated progeny were performed using *tic236-1/+* and *tic236-4/+* heterozygous plants. The self-pollinated progeny of the *tic236/+* plants exhibited ratios of 114:235:0 and 127:255:0 (wild type: heterozygous: homozygous, respectively), which are close to 1:2:0. These ratios deviated significantly from the classic Mendelian segregation ratio of 1:2:1 (**Table 1**), indicating that homozygous mutants could not be produced. These data also suggest that the gametophyte transmission efficiency is normal in *tic236/+* plants.

**Table 1:** Segregation of the *tic236-1/+* and *tic236-4/+* mutants.

Parental genotype	Progeny Genotype				Expected
Female × Male	*+/+*	*+/-*	*-/-*	*χ^2^*	*χ^2^*
*tic236-1/+* ⊗	114 (1)	235 (2.06)	0	109.10**↑**	5.99
*tic236-4/+*⊗	127 (1)	255 (2)	0	127.34**↑**	5.99

Segregation of self-progeny were performed between *tic236-1/+* and *tic236-4/+* plants. χ^2^ was chi-square test (*df* = 2, *P* < 0.05, *X^2^* > 5.99).

↑ represents the actual *Χ^2^* is increased compared with expected *Χ^2^*.

To confirm that the observed seed abortion was caused by the disruption of *TIC236*, the putative native promoter (2052 bp upstream of the start codon [ATG]) driving the full-length genomic sequence of *TIC236* plus the 3’-untranslated region (UTR; 436 bp downstream of the stop codon [TAA]) was introduced into *tic236-1/+* plants. The transgenic lines had an increased seed setting rate and seed number (**Figures 2C, D**), and homozygous *tic236* mutants were identified among the progeny (**Supplementary Figure 2C**). Thus, the mutant phenotype of *tic236/+* was caused by the deletion of *TIC236*.

### Cell division in *tic236* embryos is delayed from the octant stage

**Figure 3:**
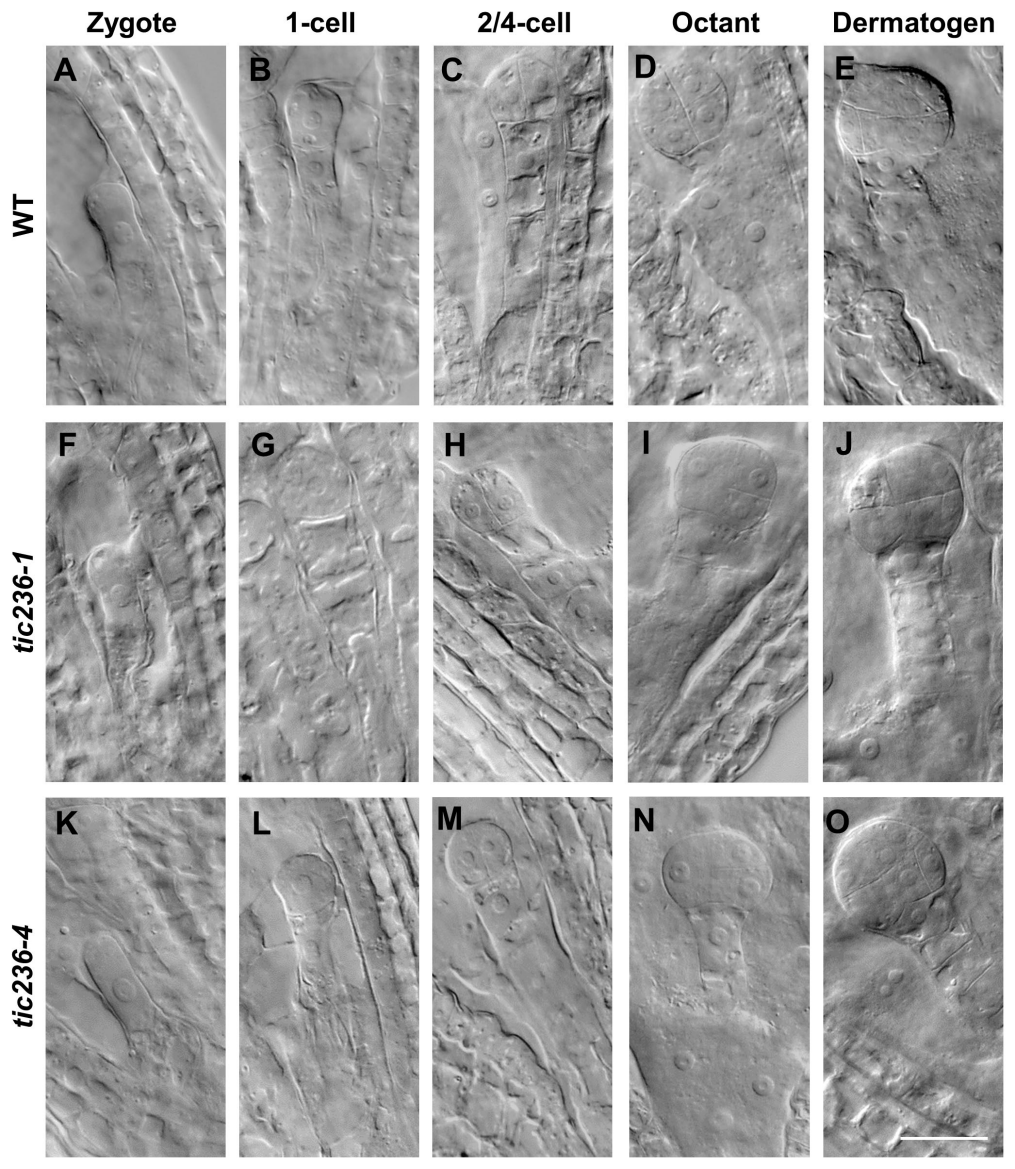
The embryo development of wild type and *tic236* mutants from zygote to dermatogen stage. **(A, F, K)** Wild-type, *tic236-1* and *tic236-4* embryos in zygote stage. **(B, G, L)** Wild-type, *tic236-1* and *tic236-4* embryos in 1-cell stage. **(C, H, M)** Wild-type, *tic236-1* and *tic236-4* embryos in 2/4-cell stage. **(D)** Wild-type embryo in octant stage. **(E)** Wild-type embryo in dermatogen stage. **(I, N)** Abnormal octant embryos in *tic236-1* and *tic236-4.* **(J, O)** Abnormal dermatogen embryos in *tic236-1* and *tic236-4*. Bar = 20 μm.

To understand the genesis of the embryo-lethal phenotype in *tic236*, we studied embryo development using siliques from WT, *tic236-1/+*, and *tic236-4/+* plants by whole-mount clearing and differential interference contrast microscopy. In wild type, elongated zygotes with obvious nuclei appeared along the future apical-basal axis (**Figure 3A**; **Supplementary Figure 3A**), and the first asymmetric division (**Figure 3B**; **Supplementary Figure 3B**) formed a smaller apical cell and a larger basal cell. At the 2/4-cell stages, two rounds of longitudinal division occurred at right angles to each other, causing the smaller apical cell to divide into four equal-sized cells (**Figure 3C**; **Supplementary Figure 3C**). The larger basal cell divided transversely to form the suspensor connecting the embryo and mother tissue (**Figure 3C**; **Supplementary Figure 3C**). In the *tic236-1* and *tic236-4* embryos, there was no obvious difference as compared with wild type from the zygote to the 2/4-cell stage (**Figures 3F–H, K–M**; **Supplementary Figures 3F–H, K–M**).

At the octant stage, a transverse division separated the embryo into upper and lower tiers creating eight cells in wild type (**Figure 3D**; **Supplementary Figure 3D**). However, half of the embryonic cells in *tic236-1* and *tic236-4* exhibited delayed transverse division, resulting in asynchronous division in the partial embryos (**Figures 3I, N**; **Supplementary Figures 3I, N**). At the dermatogen stage, a tangential division occurred in the embryo proper producing eight inner cells and eight outer cells in wild type (**Figure 3E**; **Supplementary Figure 3E**). In comparison, in *tic236-1* and *tic236-4*, the division of the embryo into upper and lower tiers occurred at this stage, but the subsequent tangential division was delayed again. Overall, tangential division occurred only in half of the cells, and only two inner cells and two outer cells were obtained in the partial embryos of *tic236-1* and *tic236-4* (**Figures 3J, O**; **Supplementary Figures 3J, O**).

**Figure 4:**
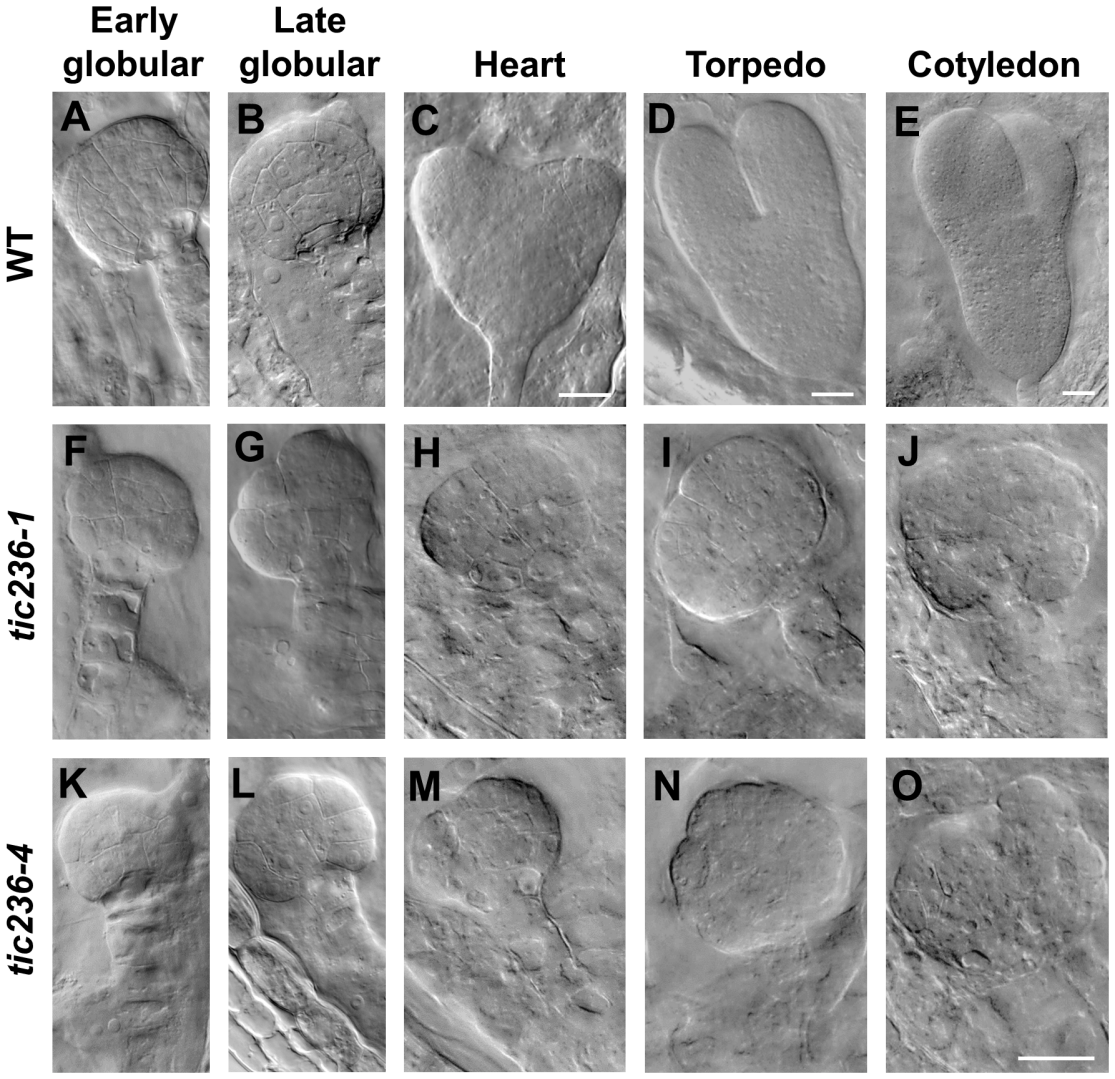
The embryo development of wild type and *tic236* from early globular to cotyledon stage. **(A–E)** Wild-type embryo in early globular **(A)**, late globular **(B)**, heart **(C)**, torpedo **(D)**, cotyledon **(E)** stages. **(F–J, K–O)** *tic236-1* and *tic236-4* embryos from siliques at different development stage as similar as wild-type embryos showed in **(A–E)**. The scale bars of **(A, B, F–O)** are the same. Bars = 20 μm.

At the early globular stage in wild type, the eight inner cells divided longitudinally while the eight outer cells underwent anticlinal divisions (**Figure 4A**; **Supplementary Figure 4A**). In *tic236-1* and *tic236-4*, the partial outer cells underwent anticlinal division, but longitudinal division was rare among the inner cells in *tic236-1* (**Figure 4F**; **Supplementary Figure 4F**). Longitudinal division was observed, however, in the lower-tier inner cells in *tic236-2* (**Figure 4K**; **Supplementary Figure 4K**). At the late globular stage, the two lower tiers of inner cells divided transversely and the outer cells underwent an additional anticlinal division to expand the outer layer in wild type (**Figure 4B**; **Supplementary Figure 4B**). Meanwhile, in the partial embryos of *tic236-1* and *tic236-4*, only a few outer cells underwent an additional anticlinal division and the inner cells did not divide transversely or longitudinally (**Figures 4G, L**; **Supplementary Figures 4G, L**). Subsequently, the WT embryos progressed through the heart, torpedo, and cotyledon stages to form bilaterally symmetrical embryos (**Figure 4C–E**; **Supplementary Figures 4C–E**). In contrast, in the siliques of *tic236-1* and *tic236-4*, embryos that remained in the defective globular stage were noted. These abnormal embryos continued to grow, but the plane of cell division was unclear leading to a more extreme aberrant morphology (**Figures 4H–J, M–O**; **Supplementary Figures 4H–J, M–O**). Together, these results show that the mutant embryos experienced delayed cell division beginning at the octant stage and reduced cell division thereafter destroying the bilateral symmetry of the embryo and arresting its development at the globular stage.

A statistical analysis of representative numbers of each of the embryonic morphologies was performed at different development stages using WT, *tic236-1/+*, and *tic236-4/+* plants (**Table 2**). Siliques were marked consecutively beginning at 1 DAP, and samples from 1 to 8 DAP were observed (Deng et al., 2014). At 1 DAP, the WT and mutant embryos contained zygotes and 1-cell stage embryos, and no significant differences were seen in the number of embryos at each development stage or in embryo division. At 2 DAP, zygote, 1-cell, 2/4-cell, and octant stage embryos were observed in the WT and mutant siliques, but the proportions of impaired octant embryos were 3.66% and 3.23% in the *tic236-1/+* and *tic236-4/+* mutants, respectively. At 3 DAP, embryos in the 2/4-cell to late globular stages were observed in wild type and the mutants, whereas the proportions of defective octant, dermatogen, and early globular stage embryos showing asynchronous division increased to 9.41% and 10% in *tic236-1/+* and *tic236-4/+*, respectively. At 4 DAP, embryos in the 2/4-cell to early heart stages were found in wild type and the mutants, but defective octant, dermatogen, early globular, and late globular stage embryos with disordered division represented 9.54% and 9.62% of the embryos in *tic236-1/+* and *tic236-4/+*, respectively (**Table 2**).

**Table 2:** Distribution of development stages in the wild type and mutants from 1 to 4 DAP siliques.

Parents	DAP	Nts/ Nsi	Nas	zygote	1 Cell	2/4 Cell	Octant	Dermatogen	EG	LG	DE	EH	Percentage of defective embryo
WT	1	275/6	45.8	45.3	0.5	—	—	—	—	—	—	—	—
*tic236-1/+*	1	320/6	53.3	52.3	1	—	—	—	—	—	—	—	—
*tic236-4/+*	1	307/6	51.2	50.5	0.7	—	—	—	—	—	—	—	—
WT	2	314/6	52.3	0.7	3.5	28.3	19.8	—	—	—	—	—	—
*tic236-1/+*	2	344/6	57.3	0.7	3.8	32.5	18	—	—	—	2.1	—	3.66
*tic236-4/+*	2	334/6	55.7	0.3	3.2	29	21.3	—	—	—	1.8	—	3.23
WT	3	332/6	55.3	—	—	0.7	2.3	20.8	25.3	6.3	—	—	—
*tic236-1/+*	3	338/6	56.3	—	—	0.8	1	17.5	24.4	7.3	5.3	—	9.41
*tic236-4/+*	3	330/6	55	—	—	1.2	3.7	16.5	20.1	8	5.5	—	10.00
WT	4	323/6	53.8	—	—	0.4	0.6	6.3	22.6	21.9	—	2	—
*tic236-1/+*	4	302/6	50.3	—	—	0.6	2	1.3	13.6	23.3	4.8	4.7	9.54
*tic236-4/+*	4	312/6	52	—	—	1	1	2	10.3	28	5	4.7	9.62

The siliques of 1 to 4 day after natural pollination were marked continuously. Each embryonic development stage of siliques from the same days after pollination was observed and counted. Each embryonic development stage displays the average of six samples. The red values highlight the number of defective embryos with disordered division and percentage of defective embryo per silique. DAP, Day after pollinate; Nts/Nsi, Number of total ovules/number of siliques; Nas, number of average ovules in a single silique; WT, wild type; EG, early globular; LG, later globular; DE, defective embryo; EH, early heart.

At 5 DAP, when the WT embryos were in the early heart or torpedo stage, about 25% of the *tic236/+* embryos were arrested at an earlier stage of development (e.g., abnormal globular stage embryos or, rarely, normal 2/4-cell and octant stage embryos), consistent with the seed abortion rates of the mutants (**Table 3**). At 6 DAP, most of the WT embryos had developed to the torpedo stage, while around 25% of the *tic236/+* mutant embryos were arrested at the abnormal globular stage or, rarely, at the normal 2/4-cell stage. At 7 and 8 DAP, when the WT embryos had reached the cotyledon stage, approximately 25% of the mutant embryos remained at the abnormal globular stage. Thus, roughly 25% of the embryos from 5 to 8 DAP in the *tic236/+* siliques were arrested at the abnormal globular embryo stage (**Table 3**).

**Table 3:** Distribution of development stages in the wild type and mutants from 5 to 8 DAP siliques.

Parents	DAP	Nts/ Nsi	Nas	2/4 Cell	Octant	Dermatogen	EG	LG	DE	EH	H	T	EC	C	Percentage of defective embryo
WT	5	273/6	45.5	—	—	—	—	—	—	8.5	25	12	—	—	—
*tic236-1/+*	5	282/6	47	0.8	0.4	—	2.3	4	11	5	17	6.5	—	—	23.40
*tic236-4/+*	5	315/6	52.5	0.5	—	—	4	7	13	2	17	9	—	—	24.76
WT	6	320/6	53.3	—	—	—	—	2.8	—	3.5	13	34	—	—	—
*tic236-1/+*	6	308/6	51.3	0.2	—	—	—	—	12.6	3.5	4	31	—	—	24.56
*tic236-4/+*	6	295/6	49.2	—	—	—	—	—	11.7	2	6.5	30	—	—	23.78
WT	7	330/6	55	—	—	—	—	—	—	—	—	1	54	—	—
*tic236-1/+*	7	289/6	48.2	—	—	—	—	—	11.2	—	—	1	36	—	23.23
*tic236-4/+*	7	308/6	51.3	—	—	—	—	—	13	—	—	0.7	37.6	—	25.34
WT	8	296/6	49.3	—	—	—	—	0.3	—	—	—	—	17	32	—
*tic236-1/+*	8	319/6	53.2	—	—	—	—	—	13.3	—	0.5	3.2	—	36.2	25.00
*tic236-4/+*	8	281/6	46.8	—	—	—	—	—	12	—	—	—	13.3	21.5	25.64

The siliques of 5 to 8 day after natural pollination were marked continuously. Each embryonic development stage of siliques from the same days after pollination was observed and counted. Each embryonic development stage displays the average of six samples. The red values highlight the number of defective embryos with disordered division and percentage of defective embryo per silique. DAP, Day after pollinate; Nts/Nsi, Number of total ovules/number of siliques; Nas, number of average ovules in a single silique; WT, wild type; EG, early globular; LG, later globular; DE, defective embryo; EH, early heart; H, heart; T, torpedo; EC, early cotyledon; C, cotyledon.

These data show that *tic236* embryos experienced delayed division, resulting in asynchronous division and a growth arrest at the globular stage, producing large, abnormal embryos with a complex morphology.

### Proplastid development and differentiation were perturbed in *tic236-4* embryos

**Figure 5:**
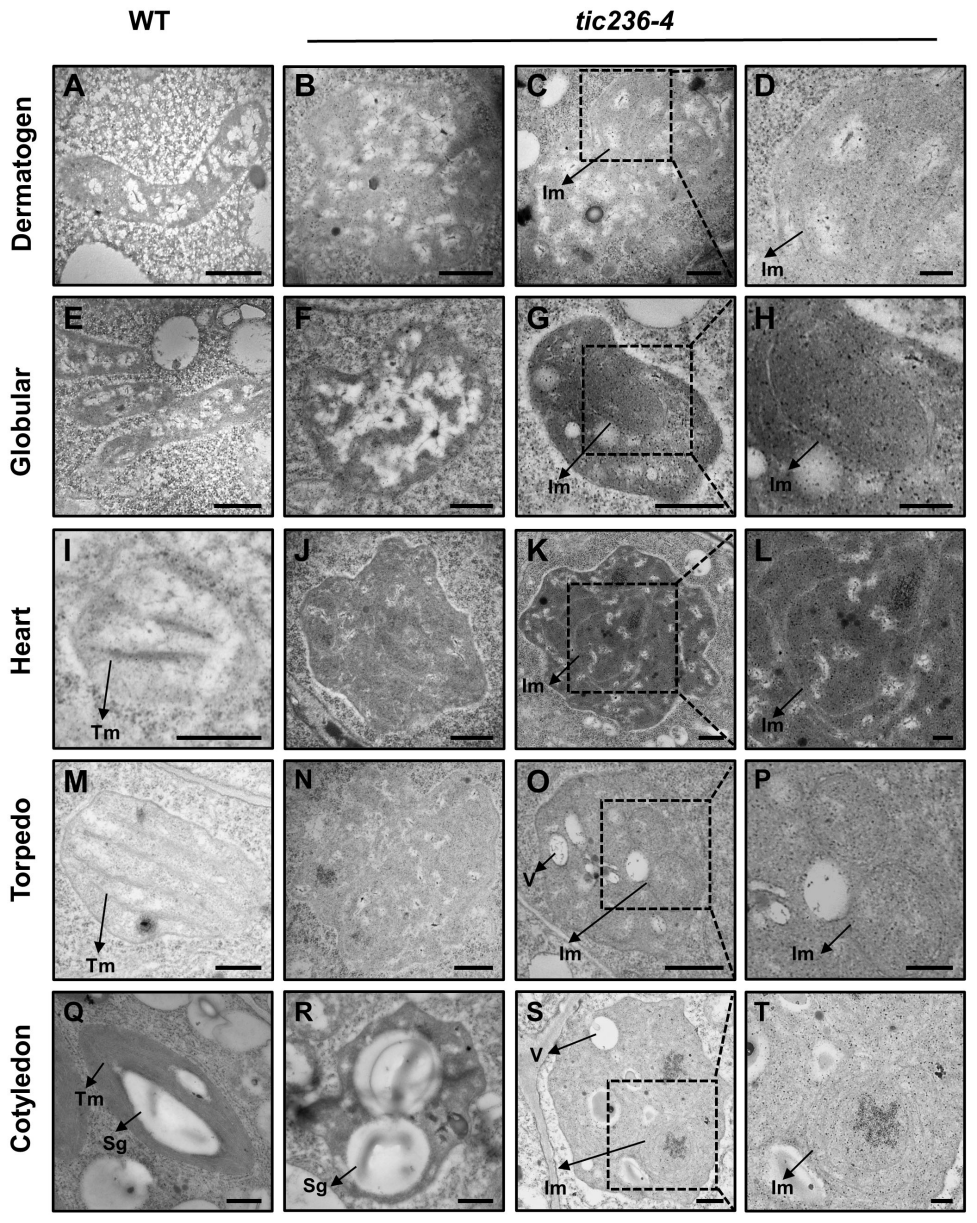
The development of proplastid is perturbed in *tic236-4* embryo cells. **(A)** Proplastid of wild type embryos in dermatogen. **(B–D)** Abnormal proplastids of *tic236-4* embryos in asymmetrical dermatogen. **(E)** Proplastid of wild type embryos in early globular. **(F–H)** Abnormal proplastids of *tic236-4* embryos in early globular. **(I)** Developing chloroplast of wild type embryos in heart. **(J–L)** Abnormal proplastids of *tic236-4* embryos in defective globular when the wild-type embryo is in heart. **(M)** Early chloroplast of WT embryos in torpedo. **(N–P)** Plastid of *tic236-4* embryos in defective globular when the wild-type embryo is in torpedo. **(Q)** Chloroplast of wild type embryos in cotyledon. **(R–T)** Abnormal plastids of *tic236-4* embryos in defective globular when the wild-type embryo is in cotyledon. **(D, H, L, P, T)** show enlarged views of the dotted boxes in **(C, G, K, O, S)**, respectively. Tm, Thylakoid membrane. Sg, Starch granules-like. Im, Internal membrane-like. V, vesicles structure. Bars = 500 nm for **(A–C, E–G, I–K, M–O, Q–S)**; 200 nm for **(D, H, L, P, T)**.

TIC236 is an essential transporter located in the inner membrane of chloroplasts, and *tic236* embryos showed defects beginning at the octant stage (**Figures 3I, N**), a stage which exhibits undifferentiated plastids. We therefore speculated that proplastid development was altered in *tic236* early embryos. Thus, transmission electron microscopy (TEM) was used to track proplastid development and chloroplast differentiation from the dermatogen to the cotyledon stages in WT and *tic236-4* mutant embryos (**Figures 5A–T**). In the dermatogen and early globular stages, the WT proplastids were small and ellipsoidal with no thylakoid membranes (**Figures 5A, E**). Strikingly, the mutant proplastids were enlarged and irregular (**Figures 5B, C, F, G**; **Supplementary Figures 5A–F**), and some proplastids contained complex intermembrane structures (**Figures 5C, D, G, H**). The morphology of the proplastids was disrupted in *tic236-4* embryos during the dermatogen stage, which is associated with embryo defects. Thus, proper proplastid development plays an important role in early embryo development.

At the heart stage, WT proplastids had begun to differentiate into chloroplasts, which were nearly round and exhibited rudimentary grana (**Figure 5I**). In the abnormal globular embryos of *tic236-4*, enlarged proplastids with an irregular morphology were observed (**Figure 5J**) with electron-dense and complex internal membranes (**Figures 5K, L**). At the torpedo stage, the WT chloroplasts were nearly elliptical and possessed grana (**Figure 5M**). In contrast, the abnormal globular stage *tic236-4* embryos contained undifferentiated proplastids with an ill-defined morphology (**Figure 5N**). Additionally, some proplastids were electron-dense and had aberrant internal membranes and vesicles (**Figures 5O, P**). At the cotyledon stage, the mature WT chloroplasts were spindle-shaped with a large number of highly organized thylakoid membranes and starch granules (**Figure 5Q**). In contrast, some abnormal plastids in the *tic236-4* embryos had starch granule-like structures with no thylakoid membranes or grana (**Figure 5R**), while other abnormal proplastids resembled those in torpedo-stage embryos (**Figures 5S, T**).

In summary, proplastid development and subsequent differentiation into chloroplasts were arrested in *tic236-4* embryos. Thus, TIC236 is required for proplastid development and chloroplast biogenesis.

### The distribution and responses of auxin and the expression profiles of *WUSCHEL-LIKE HOMEOBOX5* and *SCARECROW* were impaired in *tic236* mutant embryos

**Figure 6:**
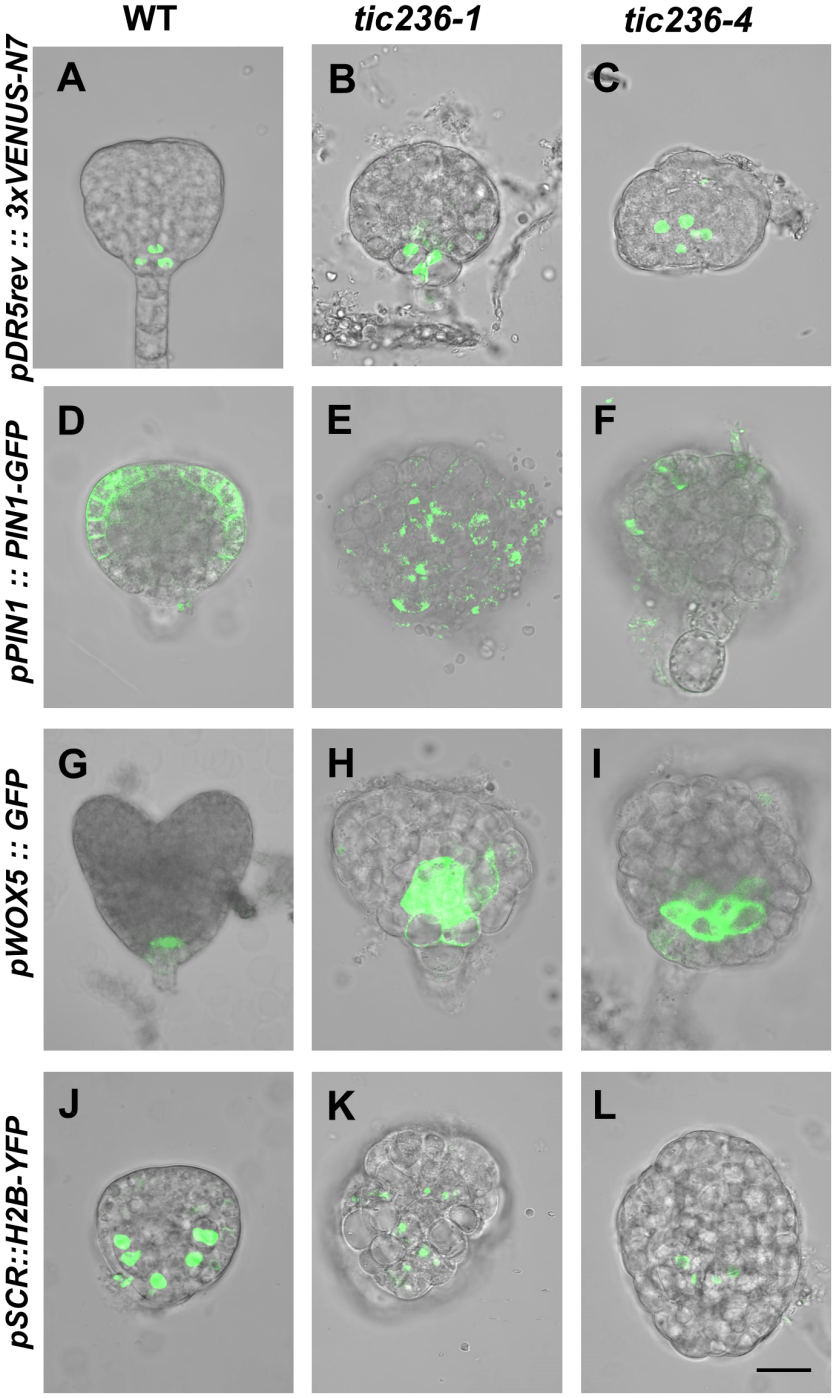
Cell-type specific expression pattern of *DR5*, *PIN1*, *WOX5*, and *SCR* were disrupted in embryos of wild type and *tic236.* **(A–C)** Auxin-responsive specific marker *pDR5rev::3xVENUS-N7* was expressed in wild type **(A)**, *tic236-1* **(B)** and *tic236-4* **(C)** globular embryos. **(D–F)** Auxin efflux transport specific marker *pPIN1::PIN1-GFP* was expressed in wild type **(D)**, *tic236-1* **(E)** and *tic236-4* **(F)**. **(G–I)** Quiescent center specific marker *pWOX5::GFP* was expressed in wild type **(G)**, *tic236-1* **(H)** and *tic236-4* **(I)**. **(J–L)** Endodermal cell layer specific marker *pSCR::H2B-YFP* was expressed in wild type **(J)**, *tic236-1* **(K)** and *tic236-4* **(L)**. Bar = 20 μm.

Auxin is involved in the establishment of bilateral symmetry and pattern formation in embryos, and an evident feature of *tic236* mutant embryos is a defect in bilateral symmetry (**Figures 4I, J, N, O**, **5G–R**) (Liu et al., 1993; Moller and Weijers, 2009; Smit and Weijers, 2015). Given that plastids are an important source of auxin precursors, the *tic236* mutation may affect auxin biosynthesis in embryos (Mano and Nemoto, 2012). We thus investigated the signal distribution of embryos carrying the *pDR5rev::3xVENUS-N7* construct which is an artificial auxin-responsive reporter (Heisler et al., 2005). In WT globular embryos, the *pDR5rev::3xVENUS-N7* signal was confined to the hypophysis (**Figure 6A**). In contrast, the distribution of *pDR5rev::3xVENUS-N7* signals in the basal or central region of the embryo proper was disorganized and the number of cells was increased in *tic236* globular embryos (**Figures 6B, C**). Therefore, auxin response was decreased in *tic236* embryos.

We also assessed the expression of the auxin polar transport carrier PIN-FORMED 1 (PIN1), which influences auxin maxima in roots and shoots (Galweiler et al., 1998; Heisler et al., 2005). In WT globular embryos, *pPIN1::PIN1-GFP* expression was restricted to the plasma membrane at the edges of the apical region (**Figure 6D**). In the mutant globular embryos, weak, diffuse, and irregular *pPIN1::PIN1-GFP* fluorescence was observed (**Figures 6E, F**). Thus, polar auxin transport via PIN1 is reduced in *tic236* embryos.

Since *tic236* embryos display impaired apical patterning, we observed the expression of *WOX5* and *SCR* (**Figures 4I, J, N, O**, **5G–R**). The *WOX5* gene encodes a homeodomain transcription factor that functions mainly in the maintenance of root stem cell identity (Heisler et al., 2005). *WOX5*, which is specifically expressed in the quiescent center (QC) in heart stage embryos, also controls auxin signaling (Blilou et al., 2005; Sarkar et al., 2007). We found that *pWOX5::GFP* signals were confined to the two cells of the QC in WT heart stage embryos (**Figure 6G**). In comparison, increased fluorescence corresponding to *WOX5::GFP* in *tic236* abnormal globular stage embryos appeared in the basal region of the embryo proper (**Figures 6H, I**). Thus, expanded expression of *WOX5* and increased QC cell division occur in *tic236* globular stage embryos.

Our *tic236* embryos exhibited defective longitudinal division of the inner cells (**Figure 4F, K**), which generate vascular and ground tissue precursors (ten Hove et al., 2015). The *SCR* gene, which is a GRAS family member, is indispensable for the periclinal division of ground tissue daughter cells leading to the formation of the endodermis and cortical cell layers during subsequent root development (Wysocka-Diller et al., 2000; ten Hove et al., 2015). *SCR* promoter-driven nuclear-localized Histon 2B (H2B) and YFP coding sequence was introduced into WT and *tic236/+* mutants to examine the *SCR* expression pattern (Heidstra et al., 2004). In WT globular stage embryos, *pSCR::H2B-YFP* signals were observed specifically in endodermal cells and were symmetrically distributed (**Figure 6J**). The same signals in globular stage embryos revealed an increased number of endodermal cells in the *tic236-1* and a reduced number of endodermal cells in the *tic236-2* which were distributed disorderly in the central region (**Figures 6K, L**). This altered expression pattern of *SCR* is consistent with the other defects seen in globular *tic236* embryos.

Therefore, auxin transport and response were significantly decreased and the expression patterns of *WOX5* and *SCR* were altered in *tic236* embryos, leading to abnormal morphogenesis of the mutant embryos.

## Discussion

Plastids, which evolved from Gram-negative cyanobacterial endosymbionts, are prominent intracellular organelles that perform various pivotal physiological and metabolic functions (Zhang et al., 2019). Embryonic proplastids are the source of plastids in plant cells (Jarvis and López-Juez, 2013). However, research on the proplastid development is very limited. Despite the prevailing belief that undifferentiated proplastids play an important role in metabolism and development, their functional roles in plant growth and development before differentiating into other types of plastids are unclear and genetic evidence supporting this view is also lacking (Jarvis and López-Juez, 2013). In addition, reports of genes that are essential for proplastid development are rare in the literature.

Biochemical analyses in Arabidopsis identified TIC236, an integral protein found in the inner membrane of chloroplasts with a cleavable plastid transit peptide (Chen et al., 2018). TIC236 associates physically with TOC75 to facilitate the translocation of preproteins into chloroplasts (Chuang et al., 2021). TIC236 and TOC75 appear to have co-evolved and may have originated from the TamB and BamA secretion machinery found in Gram-negative bacteria (Chen et al., 2018). In the present study, we found that the absence of TIC236 remarkably affected the morphology and function of embryonic proplastids, and it triggered severe abnormalities in cell division during early embryonic development. This study not only provides genetic data supporting the important roles of proplastids during early embryo development, but it also identifies *TIC236* as an essential gene required for the normal morphology and function of embryonic proplastids.

### TIC236 plays an important role in proplastid development during embryogenesis

A previous study in Arabidopsis using TEM found that plastids remain undifferentiated until the late globular stage (Mansfield, 1991). We also observed small and ellipsoidal proplastids in dermatogen and early globular stage embryos (**Figures 5A, E**). The size of the proplastids in *tic236* embryos was significantly increased (**Figures 5A–F**; **Supplementary Figures 5A–F**), and some of the proplastids contained multi-layered membrane structures (**Figures 5B–D, F–H**). TIC236 has been reported to participate in chloroplast fission by interacting with the chloroplast outer membrane protein CRUMPLED LEAF (CRL) (Fang et al., 2022). The TIC236-CRL module regulates the transport of plastic division machinery proteins into chloroplasts. Enlarged chloroplasts were observed in the leaves of plants homozygous for a weak *tic236* allele (Fang et al., 2022). A similar deficiency was found in mutants of the TIC236 homologs SSG4 in rice and DEK5 in maize. *ssg4* and *dek5* plants exhibited abnormally large chloroplasts in their leaves (Matsushima et al., 2014; Zhang et al., 2019). As embryonic cells divide, the proplastids must also divide and be distributed to the daughter cells (Moller, 2004). TIC236 may contribute to this process, controlling the division of proplastids in early embryos. Thus, the large proplastids in *tic236* plants may be due to defective division.

ARC6 also functions in proplastid division in the meristematic tissues of stems and root tips (Robertson et al., 1995). The proplastids in *arc6* plants are very long, which is different from the abnormalities observed in *tic236* proplastids. Despite the extreme delays in proplastid and chloroplast division in *arc6* plants, no obvious developmental defects occur in plant growth (Robertson et al., 1995). Therefore, defects in proplastids division may not be the only reason for the abnormal proplastids in *tic236*. TIC236 may mediate the import of a series of proteins related to the basic metabolism of proplastids. Consistent with this, disruption of the maize TIC236 homolog DEK5 affects chloroplast envelope formation, leading to a decrease in chloroplast envelope transporters (Zhang et al., 2019). It is possible that the failed TIC236-mediated transport of plastid division-related proteins and/or essential housekeeping proteins into proplastids may lead to functional and morphological defects.

There is still a lack of direct evidence to prove the localization of TIC236 in the proplastids, and marker proteins for proplastids are also not available. However TIC236 is very likely to be located in the inner membrane of the proplastid. Firstly, the proplastid also has a double-layer envelope, and the composition and function of envelope membranes between chloroplasts and proplastids are very similar (Sato, 2006). TIC236 and TOC75 are respectively homologues of bacterial inner membrane translocon TamB and outer membrane translocon BamA (Chen et al., 2018). TIC236 is present only in plants and co-evolved with TOC75 (Chen et al., 2018). Secondly, immunocolloidal gold experiments demonstrate that TIC236 mainly locates in the inner membrane of chloroplasts in leaves (Chen et al., 2018), though TIC236 is not present in TOC-TIC supercomplex in *Chlamydomonas* (Jin et al., 2022; Liu et al., 2023). The subcellular location of TIC236 should be confirmed in proplastids of early embryo in future.

### The possible function of proplastids in early embryo development

Though *TIC236* has a ubiquitous expression pattern, higher expression is found in seedling cotyledons and rosette leaves, which is consistent with role in the transport of plastid-targeted proteins and/or in plastid fission. TIC236 is also detected in developing embryos, which coincides with its function in embryonic development (**Figures 1A–J**). *tic236* embryos exhibited delayed and asynchronous cell division at the octant stage, leading to asymmetric cell division and an arrest of embryogenesis at the globular stage (**Figures 3D, E, I, G, N, O**, **4F–O**). It has been reported that mutants of key components of the plastid protein import machinery display varying degrees of defects in embryo development (Hust and Gutensohn, 2006). TIC236 interacts with the chloroplast core outer envelope translocon component TOC75 and the inner envelope translocon component TIC110 to aid the transport of plastid proteins into chloroplasts. Defects appear in *toc75* and *tic110* mutant embryos at the 2/4-cell and dermatogen stages, respectively, when the plastids are undifferentiated (Kovacheva et al., 2005; Hust and Gutensohn, 2006). Hence, TOC75 and TIC110 probably also contribute to normal proplastid function in early Arabidopsis embryos. In addition, *tic236* and *tic110* plants possess raspberry embryos during late embryo development (**Figure 4F–O**). Vacuolization, which is a key feature of cell maturation, occurs in *tic110* mutant raspberry embryos (Yadegari et al., 1995; Kovacheva et al., 2005). Therefore, we suggest that the cells in *tic236* and *tic110* embryos mature prematurely and thus do not differentiate further during the next growth stage.

How do the aberrant proplastids in *tic236* affect embryonic cell division? There are several possibilities. First, it was previously believed that proplastids, though they are undifferentiated, might provide important materials for cell growth and division. Recent transcriptome and membrane proteome data support the idea that proplastids are metabolically active organelles (Dalal et al., 2018). Proplastids supply essential metabolic precursors and export metabolic products to rapidly dividing cells (Bräutigam and Weber, 2009). Thus, the abnormal proplastids in *tic236* embryos may cause a deficiency in crucial metabolites, delaying entry into the cell division cycle or reducing cell division activity. Secondly, the asynchronous tangential division of *tic236* initially presents at the octant stage. The tangential division along the apical-basal axis, which is an important division for the generation of the two cell populations, the protoderm and the inner cells, is critical for octant embryo development (ten Hove et al., 2015). Auxin signaling is implicated in this process, and the inhibition of auxin response perturbs the tangential division (ten Hove et al., 2015). Plastids are important places for auxin biosynthesis. The synthesis of tryptophan, which is the precursor of IAA, occurs within plastids (Mano and Nemoto, 2012). Therefore, the abnormal plastids in *tic236* may influence auxin biosynthesis and/or auxin signaling. We found DR5rev::3xVENUS-N7 signal indicating auxin response is weakened and abnormally distributed in the *tic236* embryos (**Figures 6A–C**). The PIN1 expression is also remarkably reduced, which may attenuate the auxin transport and signaling (**Figures 6D–F**). Moreover, the expression of *WOX5* in QC depends on the feedback loop of WOX5-mediated auxin production and IAA17-dependent repression of auxin responses in roots (Ding and Friml, 2010; Tian et al., 2014). Accordingly, the *WOX5* expression is significantly disordered and QC did not appear in *tic236* embryo (**Figures 6G–I**). Thus, the delayed cell division of *tic236* embryo may be partially due to compromised auxin signaling. Thirdly, during embryonic cell division, proplastids must divide and be distributed to daughter cells (Moller, 2004). It has been reported that the giant chloroplasts caused by the mutation of the CRL protein disrupt the cell cycle and elevate endoreduplication activity resulting in retarded growth, possibly due to retrograde signaling from the chloroplasts back to the nucleus (Hudik et al., 2014; Fang et al., 2022). Therefore, the large proplastids in *tic236* embryos may trigger abnormal cell division through retrograde signaling to the nucleus. Last but not least, numerous works have demonstrated chloroplast biogenesis in embryos is crucial for embryo development (Lu et al., 2014; Li et al., 2021). Proplastids in *tic236* embryos cannot successfully differentiate into the chloroplast, which may further aggravate the retarded division.

Collectively, our findings discover that TIC236 promotes embryonic proplastid development and differentiation, and provide genetic evidence supporting the important role of proplastid development in plant embryogenesis.

## Materials and methods

### Plant materials and growth conditions

The *Arabidopsis thaliana* plants used in this study were of the Columbia ecotype. The T-DNA insertion mutants *tic236-1* (SALK_048770) and *tic236-4* (SALK_149862C) were obtained from the ABRC (http://www.arabidopsis.org) (Alonso et al., 2003). *pDR5rev::3XVENUS-N7* and *pPIN1::PIN1-GFP* (Heisler et al., 2005) were obtained from Elliot Meyerowitz (Division of Biology, California Institute of Technology, CA, USA). *pDR5rev::3xVENUS-N7* consists of nine repeats of the auxin-response element (TGTCTC) fused in inverse orientation to the CaMV minimal 35S promoter, the 3xVENUS coding sequence and N7 nuclear-localization sequence (Ulmasov et al., 1997; Friml et al., 2003; Heisler et al., 2005). “rev” means “reverse”. *pSCR::H2B-YFP* and *pWOX5::GFP* lines (Heidstra et al., 2004; Blilou et al., 2005) were obtained from Ben Scheres (Department of Biology, University of Utrecht, The Netherlands). These reporter lines were crossed with *tic236-1*/+ and *tic236-4/+*.

Seeds were sterilized in 75% ethanol, rinsed three times with sterile water, and then planted on MS medium (4.33 g/L of Murashige and Skoog basal salt mixture with 0.75% agar and 2% sucrose, pH 5.75). The MS plates were placed at 4°C for 3 days and then transferred to growth chambers under long-day conditions (16 h of light/8 h of darkness) at 22°C. After the seedlings had grown for 10 days on MS medium, they were transplanted to soil for growth in a greenhouse (22°C, long-day conditions).

### Genotyping analysis

Genomic DNA was extracted from leaves as described previously (Edwards et al., 1991). The T-DNA insertions were analyzed by PCR genotyping with the specific primers *tic236-1*-FP/RP and *tic236-4*-FP/RP. LBb1.3 was used as a common primer for T-DNA detection. The primers are listed in **Supplementary Table 1**.

### Plant transformation and selection

All plants were transformed via *Agrobacterium tumefaciens* (strain GV3101)-mediated floral-dip transformation (Clough and Bent, 1998) and selected on MS medium containing 25 mg L^-1^ of hygromycin B (Calbiochem, San Diego, CA, USA).

### Molecular cloning

To analyze *TIC236* expression, a genomic DNA fragment containing 2054 bp upstream of the start codon [ATG] of the *TIC236* was amplified by PCR from genomic DNA of Arabidopsis ecotype Col-0 plants using specific primers named *TIC236 pro-GUS-FP/RP*. The amplified DNA fragment was verified by sequencing and cloned into the *pCambia1300-Pro35S::GUS* binary vector using *Sal*I/*Bam*HI restriction sites. The promoter of *TIC236* replaced the *35S* promoter by One Step Cloning Kit (support@vazyme.com). *pCambia1300-ProTIC236::GUS* cloning was obtained and transformed into a wild-type plant. The transgenic seeds were selected on MS medium containing 25 mg L^-1^ of hygromycin B.

A *TIC236* genomic DNA fragment including 2054 bp of the promoter and 436 bp of the 3′-UTR was cloned from wild-type genomic DNA using specific primers *TIC236*-genome-FP/RP (**Supplementary Table 1**). The amplified DNA fragment was verified by sequencing and cloned into pCambia1300 binary vector using *Bam*HI/*Kpn*I by One Step Cloning Kit (support@vazyme.com) which was transferred into a *tic236-1/+* mutant plant. After screening the transgenic seeds on hygromycin plates, positive transformants were identified by PCR using specific primers *tic236-1*-*Comp*-FP/RP (**Supplementary Table 1**) and then used for subsequent analysis.

### qRT-PCR

Total RNA was isolated using an Eastep Super Total RNA Extraction Kit (LS1040; Promega, Madison, WI, USA). The cDNAs were synthesized from 1 μg of total RNA using a Prime Script RT Reagent Kit (RR037; Takara Bio Inc., Ostu, Japan) according to the manufacturer’s instructions.

qRT-PCR was performed using the Bio-Rad qRT-PCR System (Hercules, CA, USA) and Ultra SYBR Mixture (CW0957M; CWBIO, Cambridge, MA, USA). ACTIN7 served as an internal control. The expression of all sample was normalized to the sample “1 DAP siliques” expression. The 2^-ΔΔct^ method was used to calculate the expression levels. Three independent biological and technological replicates were tested for each sample. Bio-Rad CFX Manager and GraphPad Prism 5 were used to analyze the data. The primers used for qRT-PCR are shown in **Supplementary Table 1**.

### GUS staining

The construct *p1300*-*ProTIC236:GUS* was transformed into WT plants. GUS activity was analyzed by staining various tissues in a solution of 1 mg/mL of X-gluc (Goldbio, St. Louis, MO, USA), 10 mM EDTA·2Na, 0.5 mM K_3_FeC_6_N_6_·3H_2_O, 50 mM NaH_2_PO_4_, 50 mM Na_2_HPO_4_, and 0.1% (v/v) Triton X-100 at 37°C until they were appropriately stained. Next, the chlorophyll was removed using absolute ethanol and the tissues were treated with 2% HCl (v/v) in 20% methanol (v/v) and 7% NaOH (w/v) in 60% ethanol (v/v). After that, we rehydrated the materials using 60%, 40%, 20%, 10%, and 5% ethyl alcohol. The tissue staining was observed under a digital microscope (DVM6, Leica, Germany) and the embryo staining was examined under a microscope (AXIO Imager A1, Zeiss) after adding 50% glycerin.

### eFP browser data

The expression map of *TIC236* was obtained from the eFP Browser (http://bar.utoronto.ca/eplant/; Nakabayashi et al., 2005; Schmid et al., 2005; Winter et al., 2007).

### Observation of embryo development

To track embryo development, open and flat flowers from WT and mutant plants were marked continuously with colorful thread at 10 AM for 8 days. The marked siliques were collected and the carpels were cut open using a dissecting needle under a stereomicroscope. Then, the marked siliques were placed in fixation solution (4% glutaraldehyde and 0.1% Triton X-100 in PBS, pH 7.4) under negative pressure using a vacuum pump overnight. After that, each sample was transferred to a clearing solution [chloral hydrate:water:glycerol at a ratio of 8:2:1 (w/v/v)] for minutes to hours depending on the developmental stage of the embryo. Ovules with embryos were dissected from the siliques and the embryos were visualized using differential interference contrast microscopy (AXIO Imager A1; Zeiss, Jena, Germany).

### Detection of fluorescence in the embryos

For fluorescent marker line analysis, WT and mutant globular embryos were isolated from ovules and mounted in 10% (v/v) glycerol. Fluorescent signals were visualized using an FV3000 confocal laser scanning microscope (Olympus, Tokyo, Japan). GFP/YFP/3xVENUS fluorescence was detected with excitation at 488 nm.

### Transmission electron microscopic observation

For ultrastructural analysis, WT and *tic236-1/+* mutant ovules at 2 DAP, 3 DAP, 5 DAP, and 7 DAP were fixed with 2.5% glutaraldehyde in 50 mM sodium phosphate buffer (pH 7.4). The samples were treated with 1% osmium tetroxide in 50 mM sodium phosphate buffer (pH 7.4) for 3 h and embedded in Spurr’s resin as described elsewhere (Sanders et al., 1999). Ultrathin sections were stained with saturated uranyl acetate and lead citrate and examined with a transmission electron microscope (H-7650; Hitachi, Tokyo, Japan).

## Data Availability

The original contributions presented in the study are included in the article/**Supplementary Material**, further inquiries can be directed to the corresponding authors.
