# Supplementary material for: Arabidopsis TIC236 contributes to proplastid development and chloroplast biogenesis during embryogenesis
Source: Front Plant Sci. 2024 Aug 23;15:1424994. doi: 10.3389/fpls.2024.1424994 (PMC11377289; doi:10.3389/fpls.2024.1424994)
Supplement: Supplementary file 1 [file DataSheet1.pdf]

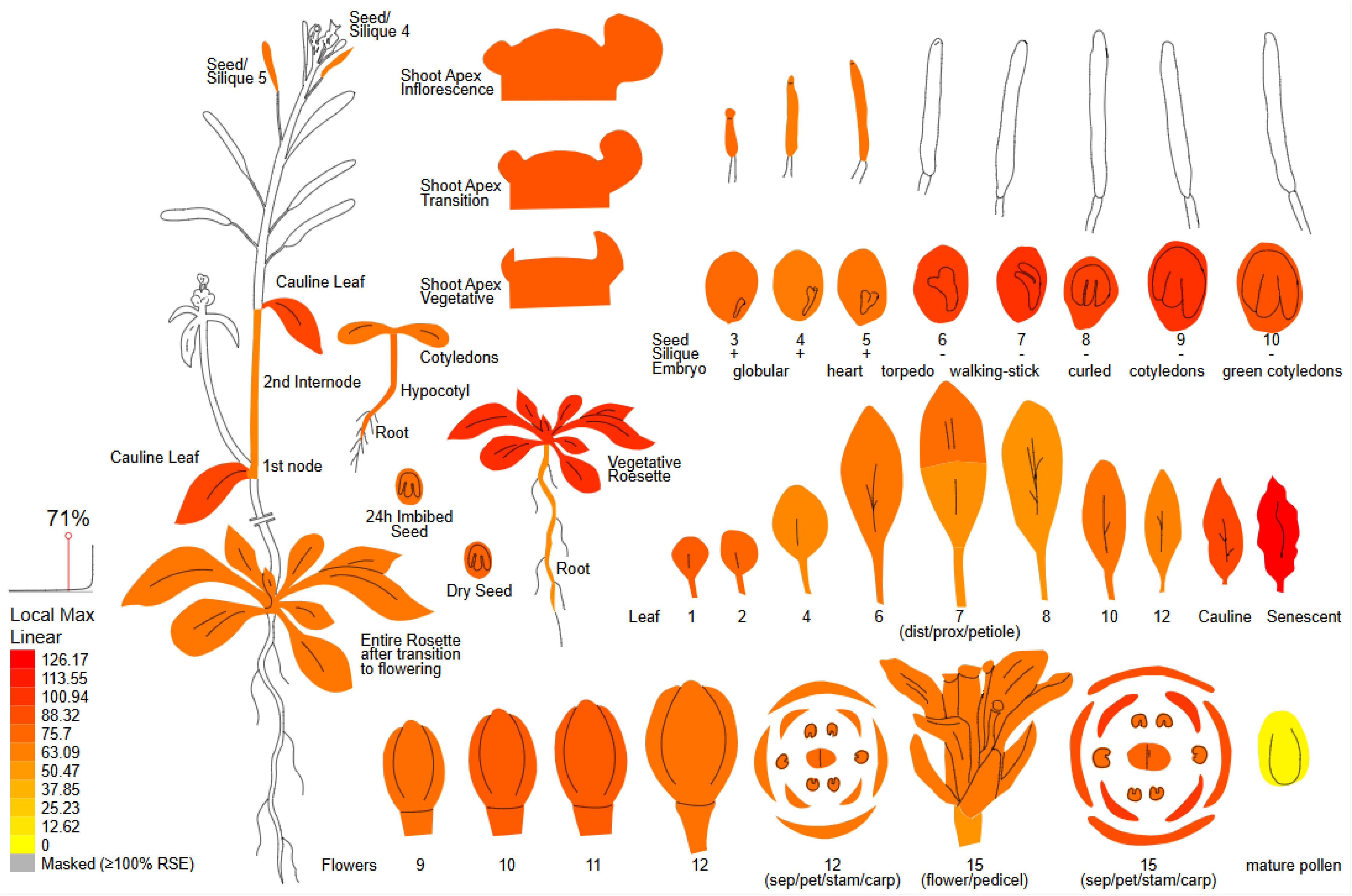

Supplemental Figure 1. Expression profile analysis of *TIC236***.** Data were obtained from the Arabidopsis eFP Browser (http://bar.utoronto.ca/eplant/).

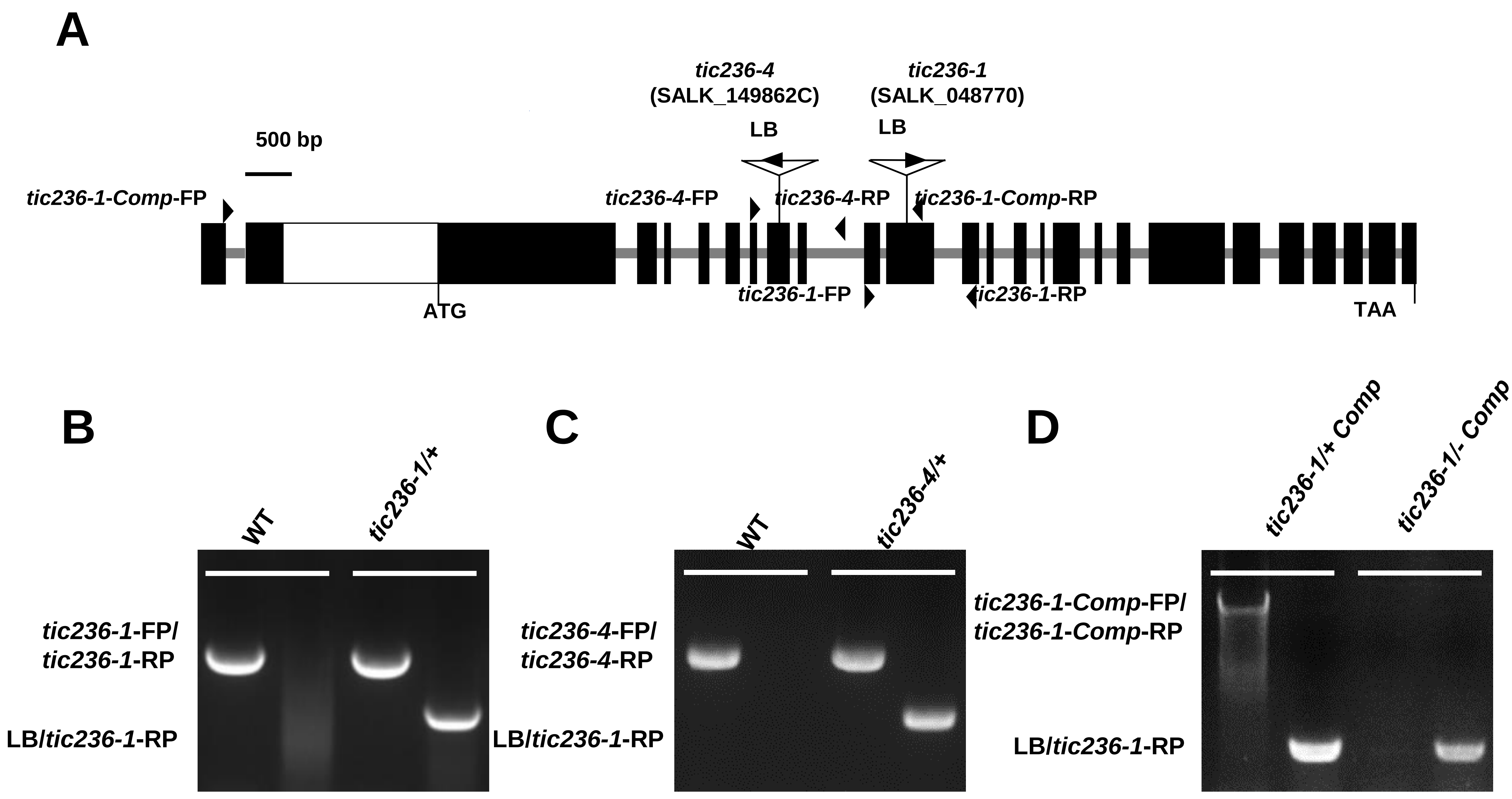

Supplemental Figure 2. Genotype analysis of *tic236* mutant and functionally complemented line. (A) Schematic diagram of the *TIC236* genomic sequence structure and each T-DNA insertion site. The primers *tic236-1*-FP/ *tic236-1*-RP and *tic236-4*-FP/ *tic236-4*-RP were designed for genotype analysis of *tic236-1*and *tic236-4*, respectively. The primer *tic236-1-Comp*-FP/ *tic236-1-Comp*-RP was designed for genotype analysis of functionally complemented (*Comp*) *tic236-1* /+ transgenic plants. Black boxes and grey lines indicate exons and introns, respectively. The white box indicates the untranslated region.

(B) PCR analysis of *tic236-1/*+ mutants.

(C) PCR analysis of *tic236-4/*+ mutants.

(D) PCR analysis of functionally complemented *tic236 -1/*+ transgenic plants.

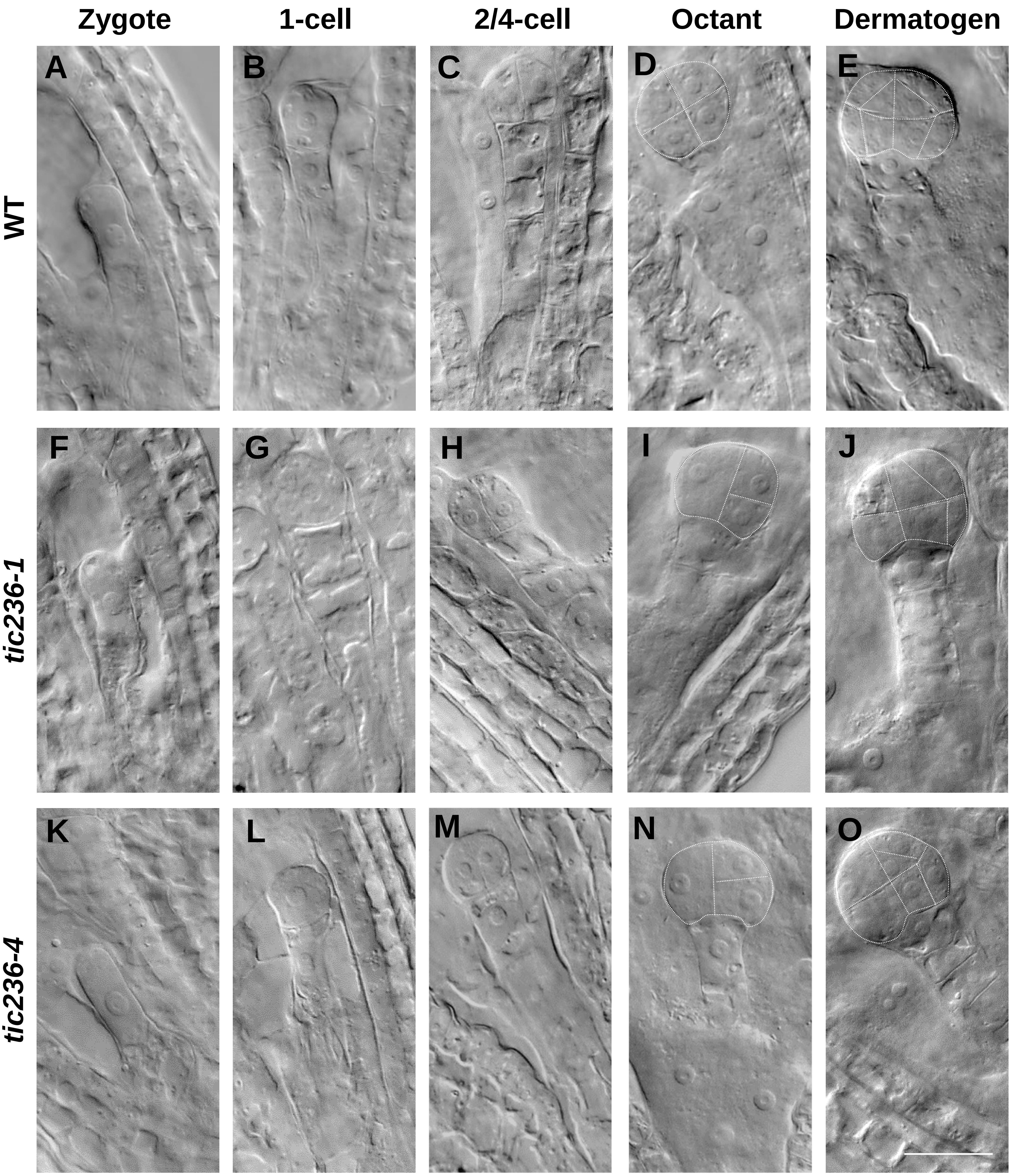

Supplemental Figure 3. The embryo development of wild type and *tic236* mutants from zygote to dermatogen stage**.**

(A), (F) and **(K)** Wild-type, *tic236-1* and *tic236-4* embryo in zygote stage.

(B), (G) and **(L)** Wild-type, *tic236-1* and *tic236-4* embryo in 1-cell stage.

(C), (H) and **(M)** Wild-type, *tic236-1* and *tic236-4* embryo in 2/4-cell stage.

(D) Wild-type embryo in octant stage.

(E) Wild-type embryo in dermatogen stage.

(I) and **(N)** Abnormal octant embryos in *tic236-1* and *tic236-4.*

(J) and **(O)** Abnormal dermatogen embryos in *tic236-1* and *tic236-4*. The white line indicates embryo proper outline and the cell division plane. Bar = 20 μm.

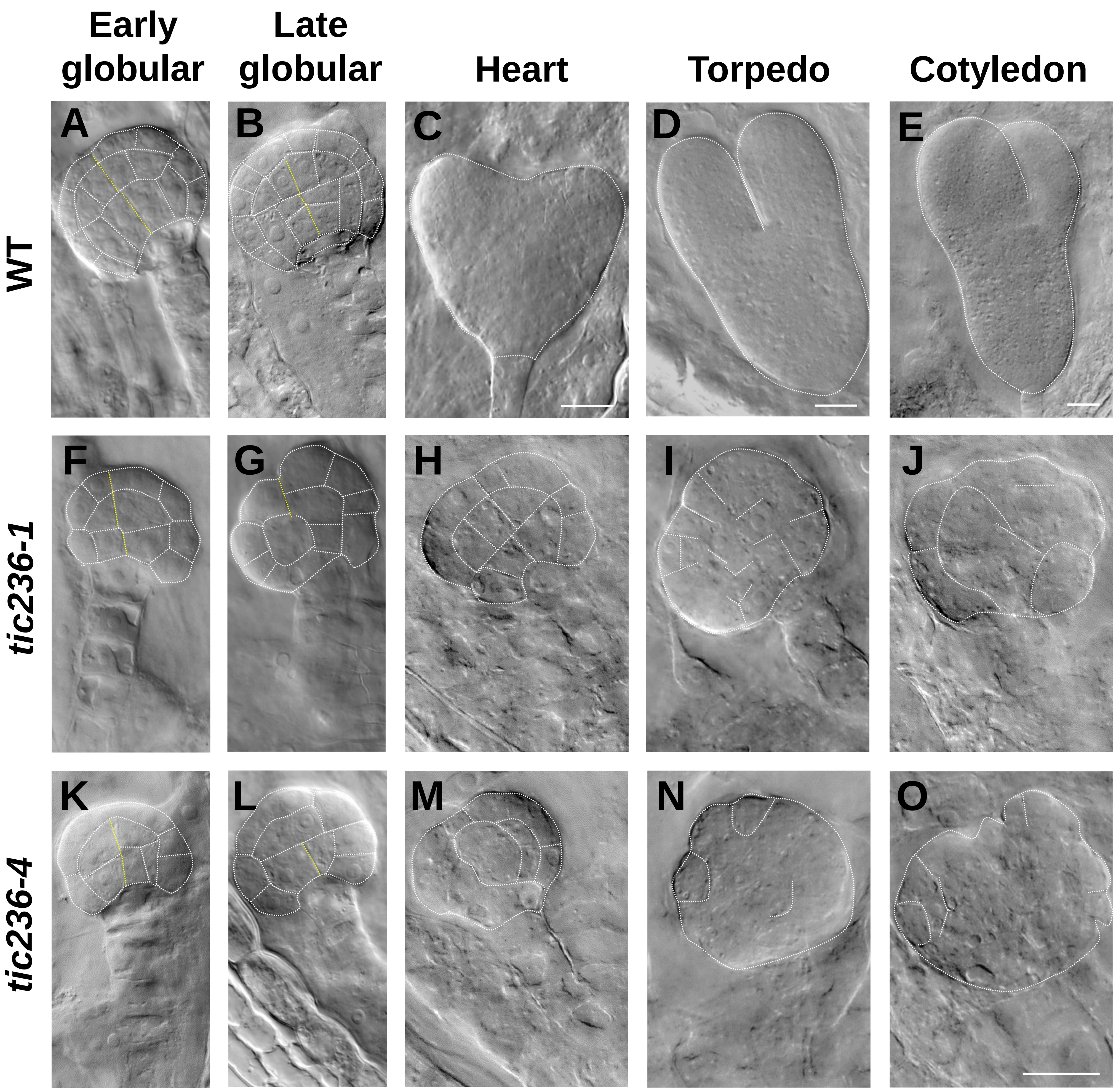

Supplemental Figure 4. The embryo development of wild type and *tic236* from early globular to cotyledon stage.

(A) to **(E)** Wild-type embryo in early globular **(A)**, late globular **(B)**, heart **(C)**, torpedo **(D)**, cotyledon **(E)** stages.

(F) to **(J)** and **(K)** to **(O)** *tic236-1* and *tic236-4* embryos from siliques at different development stage as similar as wild-type embryos showed in **(A) to (E)**. The white line indicates embryo proper outline and the cell division plane. The yellow line represents possible longitudinal division plane in the middle. The scale bars of **(A)** to **(B)** and (**F)** to **(O)** are the same. Bars = 20 μm.

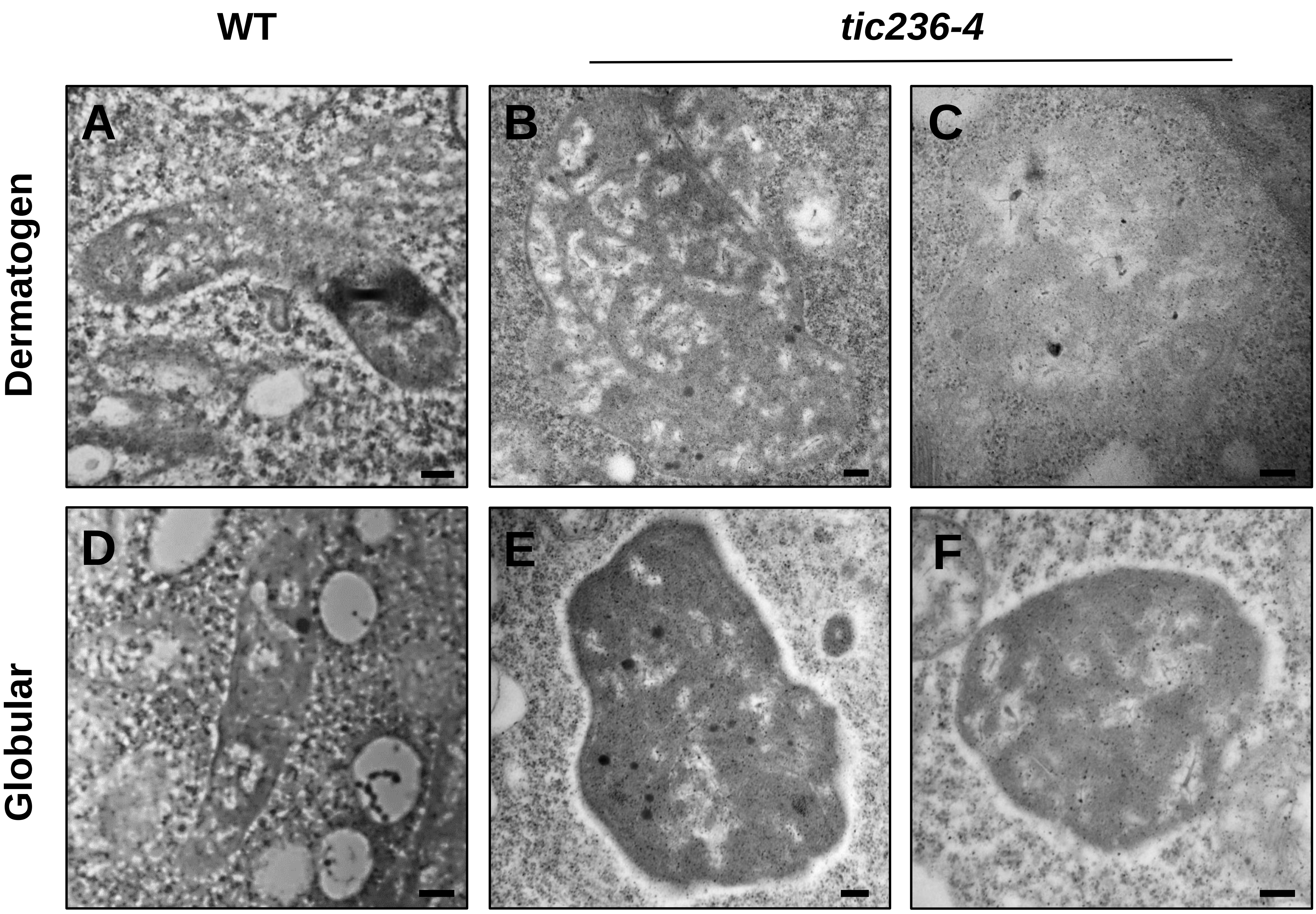

Supplemental Figure 5. TEM observation of proplastids at dermatogen and early globular embryos of wild type and *tic236-4*.

(A) and **(D)** Proplastids of wild-type embryos at dermatogen and globular stage, respectively.

(B) and **(C)** Abnormal proplastids of *tic236-4* in asymmetrical dermatogen embryos.

(E) and **(F)** Abnormal proplastids of *tic236-4* embryos at early globular stage. Bars = 200 nm.

**Supplemental Table I. Primers used for identification of T-DNA insertion mutants and constructions**		
Primer name	Sequence	Purpose
tic236-1-FP	TTTACAGGGGGAGGTGGAT	For identifying the genotype of *tic236-1* mutant plants
tic236-1-RP	AGCGTTAGCTCTTAGTTGTCCCT	
tic236-4-FP	TTCAAGGAAATGGCAATCAAC	For identifying the genotype of *tic236-4* mutant plants
tic236-4-RP	TGTTTCAGGTTCCCTATGTGG	
tic236-1-Comp-FP	GTTGCCCCCAAGGAGAAC	For identifying the genotype of *tic236-1* mutant plants in complementary assay
tic236-1-Comp-RP	CCTTCCTGTTGCTTTTAAATGTG	
LBb1.3	ATTTTGCCGATTTCGGAAC	Universal primer for genotyping of T-DNA insertion mutants
TIC236-qRT-FP	TGTCAGGTTGATACTCAGCTGC	For amplifying the endogenous transcript of *TIC236* in qRT-PCR test
TIC236-qRT-RP	GTACATTATCCTCAGCTCTGGTCC	
ACTIN7-FP	AGGCACCTCTTAACCCTAAAGC	For amplifying the endogenous transcript of *AtACTIN7* in qRT-PCR test as loading control
ACTIN7-RP	GGACAACGGAATCTCTCAGC	
TIC236-genome-FP	CAGGTCGACTCTAGAGGATCCCA GAGGCTGGACAAGAAAAGAA	For amplifying the genomic DNA in complementary assay
TIC236-genome-RP	TACGAATTCGAGCTCGGTACCGC CGTTGTAGCATTTGGTG	
TIC236 pro-GUS-FP	GCGTCGACCAGAGGCTGGACAA GAAAAGAA	For amplifying the native promoter for GUS staining
TIC236 pro-GUS-RP	CGGGATCCTATCACGTGCTCATCT GCTGG	
