# Supplementary figures and images for: Arabidopsis TIC236 contributes to proplastid development and chloroplast biogenesis during embryogenesis
Source: Front Plant Sci. 2024 Aug 23;15:1424994. doi: 10.3389/fpls.2024.1424994 (PMC11377289; doi:10.3389/fpls.2024.1424994)

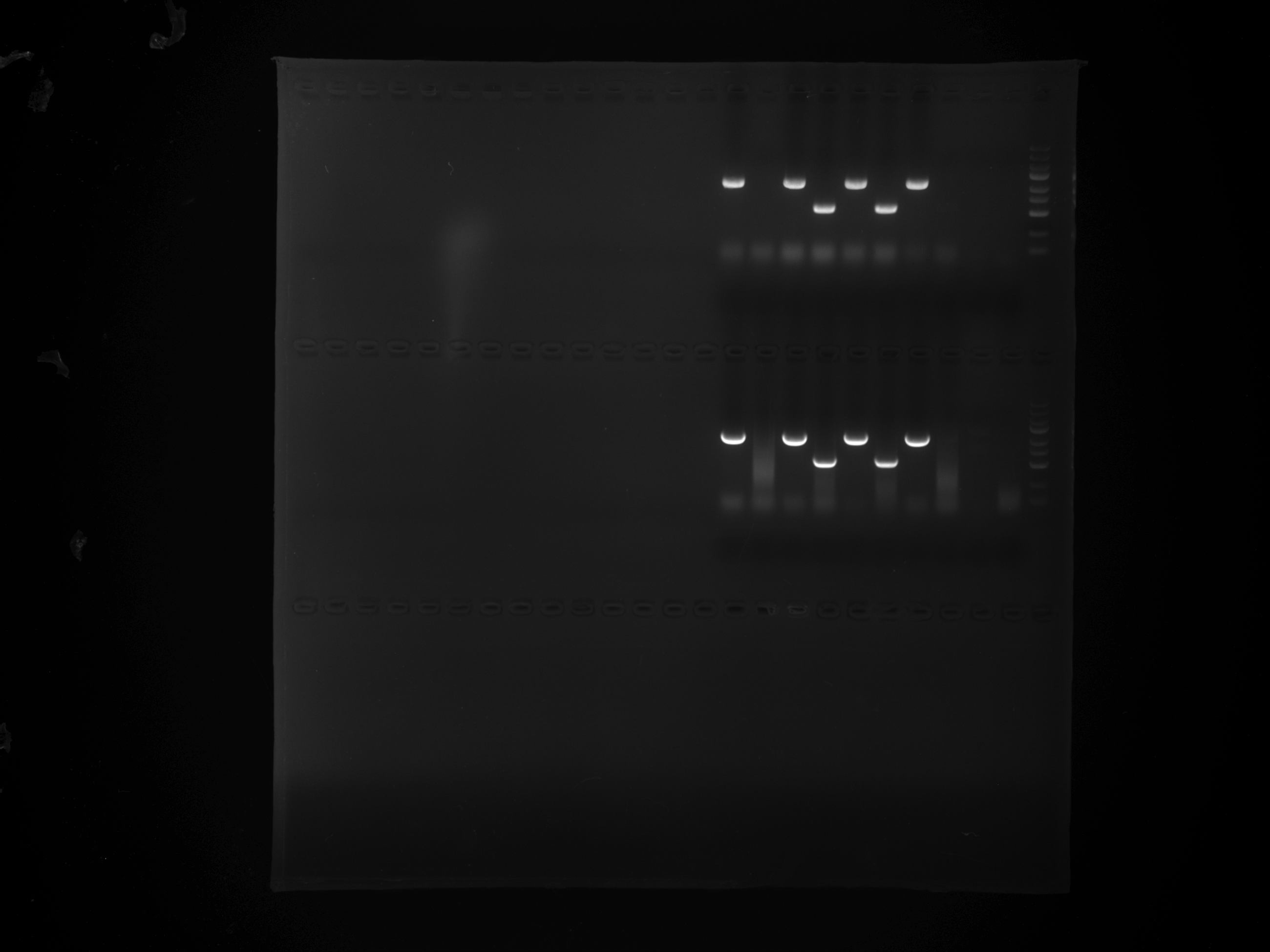

Supplement: Supplementary file 2 [file Image1.tif]

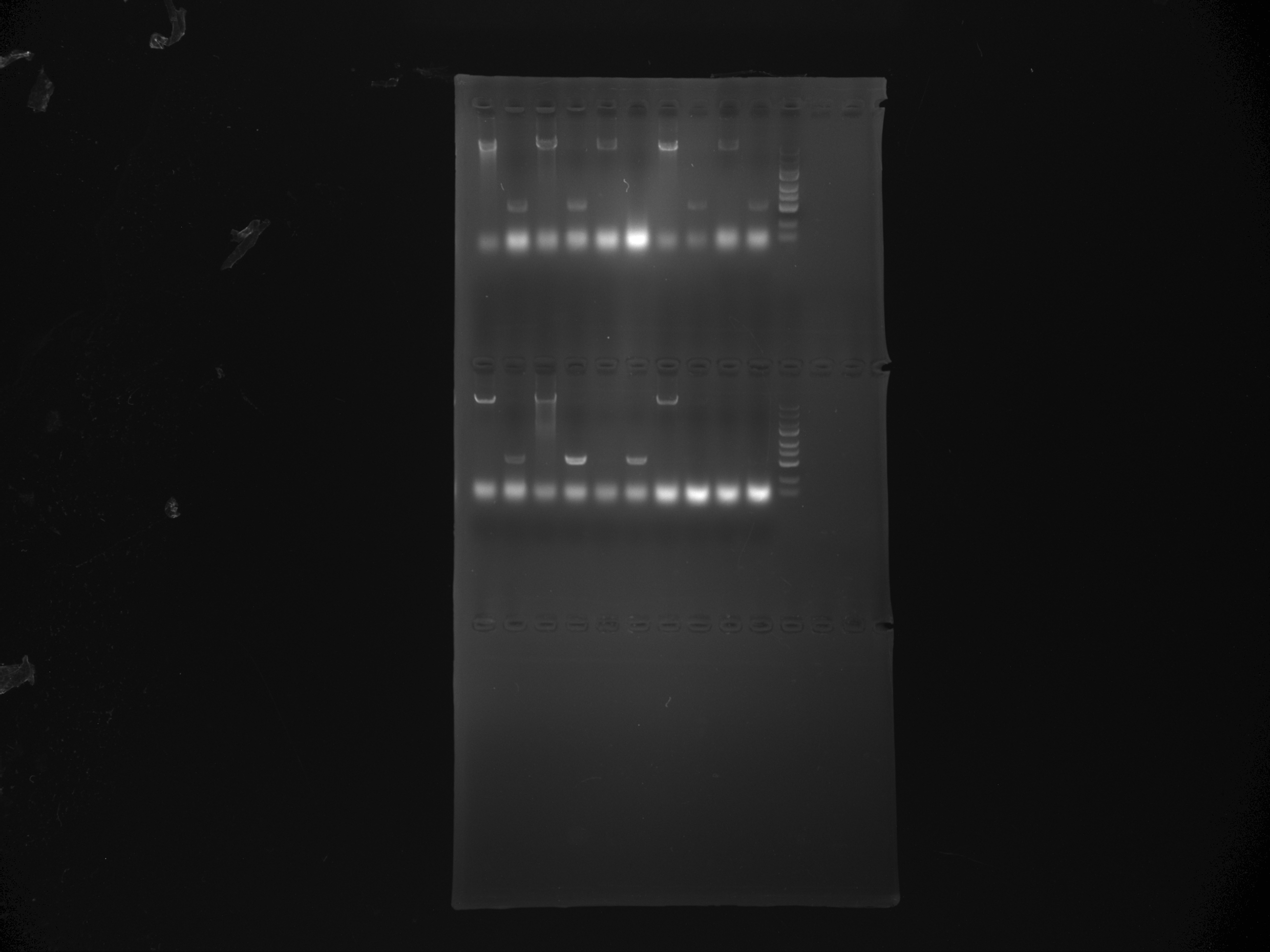

Supplement: Supplementary file 3 [file Image2.tif]
